# Selective Activation of the Spinal Cord with Epidural Electrical Stimulation

**DOI:** 10.3390/brainsci14070650

**Published:** 2024-06-27

**Authors:** Carlos Cuellar, Lauri Lehto, Riaz Islam, Silvia Mangia, Shalom Michaeli, Igor Lavrov

**Affiliations:** 1 School of Sport Sciences, Universidad Anáhuac México, Huixquilucan 52786, Mexico; carlos.cuellarra@anahuac.mx; 2 Center for Magnetic Resonance Research (CMRR), Department of Radiology, University of Minnesota, Minneapolis, MN 55455, USA; ljlehto@gmail.com (L.L.); mangia@umn.edu (S.M.); 3 Department of Neurology, Mayo Clinic, Rochester, MN 55905, USA; riazeee06@gmail.com; 4 Laboratory of Neuromodulation, Institute of Fundamental Medicine and Biology, Kazan Federal University, 420008 Kazan, Russia; 5 Department of Physiology and Biomedical Engineering, Mayo Clinic, Rochester, MN 55905, USA

**Keywords:** spinal cord stimulation, epidural stimulation, neuromodulation, functional neuroanatomy, spinal cord injury, pain, spatial-selective stimulation, orientation-selective stimulation, spinally evoked motor potentials, rats

## Abstract

Spinal cord epidural electrical stimulation (EES) has been successfully employed to treat chronic pain and to restore lost functions after spinal cord injury. Yet, the efficacy of this approach is largely challenged by the suboptimal spatial distribution of the electrode contacts across anatomical targets, limiting the spatial selectivity of stimulation. In this study, we exploited different ESS paradigms, designed as either Spatial-Selective Stimulation (SSES) or Orientation-Selective Epidural Stimulation (OSES), and compared them to Conventional Monopolar Epidural Stimulation (CMES). SSES, OSES, and CMES were delivered with a 3- or 4-contact electrode array. Amplitudes and latencies of the Spinally Evoked Motor Potentials (SEMPs) were evaluated with different EES modalities. The results demonstrate that the amplitudes of SEMPs in hindlimb muscles depend on the orientation of the electrical field and vary between stimulation modalities. These findings show that the electric field applied with SSES or OSES provides more selective control of amplitudes of the SEMPs as compared to CMES. We demonstrate that spinal cord epidural stimulation applied with SSES or OSES paradigms in the rodent model could be tailored to the functional spinal cord neuroanatomy and can be tuned to specific target fibers and their orientation, optimizing the effect of neuromodulation.

## 1. Introduction

Spinal cord epidural electrical stimulation (EES) has been successfully applied to treat chronic pain [1] and to enable sensorimotor function in patients with spinal cord injury (SCI) [2,3,4,5,6,7,8,9]. EES also demonstrated encouraging results in restoring blood pressure in patients with SCI [10,11] and bladder control [12]. The mechanisms of EES and the role of different targets are still the subject of discussion [13,14,15,16,17]. Different approaches were proposed to optimize the distribution of the electrical field across key targets, dorsal roots, and dorsal columns, by variation in the electrode number and configurations [2,3,4,18,19,20]. To cover the complex geometry of the spinal targets and increase the specificity of conventional EES, several epidural electrode arrays with high contact density were developed and demonstrated some efficacy [21,22,23]. Low spatial selectivity and lead migration [24] remain the key limitations of optimum spatial and temporal control during EES [13,25]. An approach that could provide a flexible and adjustable orientation of electrical current is highly desirable. Recently we demonstrated the critical role of the spinal cord functional neuroanatomy in the effect of EES to evoke motor-evoked responses [14] and evoked compound action potentials (ECAP) [19] and proposed a segment-specific stimulation in humans [15]. These findings suggest that a higher level of resolution and specificity of EES can be achieved with spatial-selective and orientation-selective EES. As the spatial-selective approach is limited by the density of the electrodes, recently, orientation-selective stimulation has demonstrated selectivity in the activation of the brain structures [26,27,28,29,30]. This study, for the first time, implemented and compared Spatial-Selective and Orientation-Selective Epidural Stimulation (SSES and OSES, respectively) to evoke Spinally Evoked Motor Potentials (SEMPS) using four or three contacts on a four-channel electrode.

## 2. Materials and Methods

**Figure 1:**
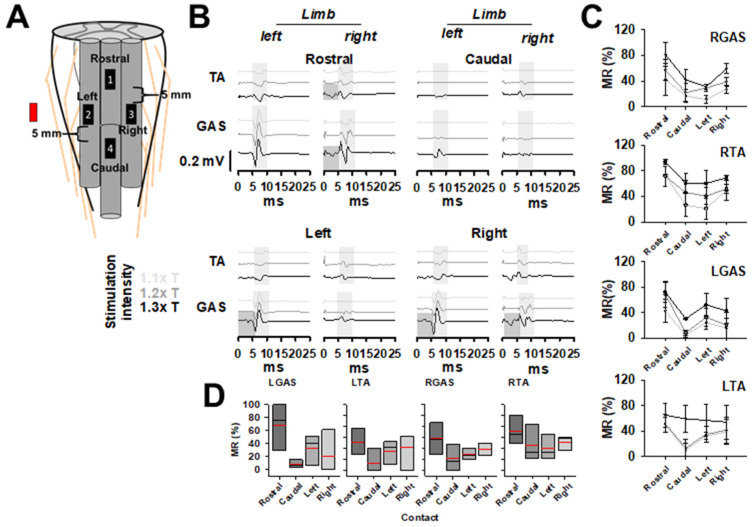
Conventional Monopolar Epidural Stimulation, CMES. (**A**) The position of the contact leads (black rectangles numbered 1, rostral; 2, left; 3, right and 4, caudal, cathodes) on the dorsal spinal cord and the relative position of the anode (red rectangle). (**B**) Bilateral TA and GAS SEMPs recorded during CMES delivered one electrode at a time in a representative experiment. Each trace is the average of ten responses obtained with increasing stimulation intensities at 1.1 xT, 1.2 xT, and 1.3 xT. (**C**) Averaged and normalized MR (mean ± SE%, *n* = 3) for bilateral TA and GAS for each contact lead during CMES at increasing stimulation intensities expressed as x times threshold for MR. MR amplitudes evoked at 1.2 xT were used to construct graphs in (**D**). Black and red lines represent the median and mean, respectively. No statistically significant differences were found when comparing MR across contact leads in any muscle. *p* > 0.05.

**Figure 2:**
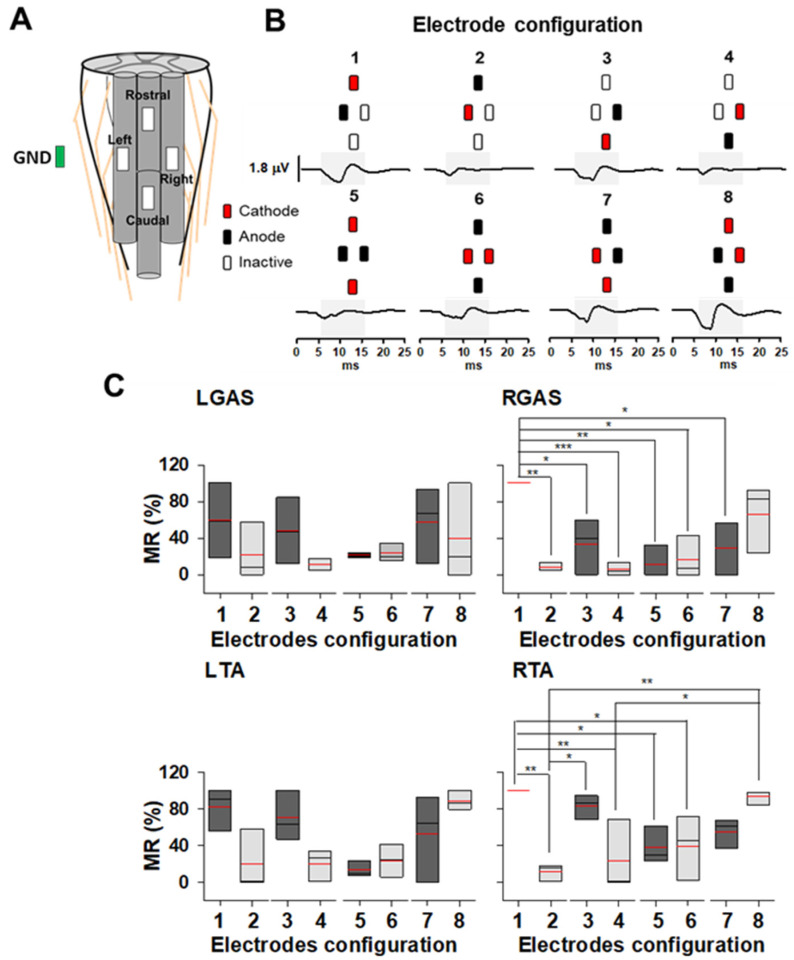
Bipolar spatial selective epidural stimulation, SSES. (**A**) The 4-contact multielectrode array was used to deliver bipolar SSES at 1.2 xT in 8 different electrode configurations numbered 1–8. The green rectangle represents the relative position of the ground (GND) electrode. (**B**) Averaged SEMPs (10 responses for each trace) in a representative experiment across bipolar SSES configurations in left TA. MR is highlighted by the light gray rectangles. Cathode (s) and anode (s) configurations are shown on top of each trace. (**C**) Normalized MR amplitude (mean ± SE%, *n* = 3) in left/right TA and GAS for each bipolar SSES configuration. Each bar plot corresponds to a configuration (1–8) as shown in (**B**). Statistical differences were found when comparing configuration 1 vs. configurations 2, 3, 4, 5, 6, and 7 in RGAS. In RTA, statistical differences were found when comparing configurations 1 vs. 2, 4, 5, and 6; 2 vs. 3, 8; and 4 vs. 8. Black and red lines represent the median and mean, respectively. * *p* < 0.05, ** *p* < 0.01, *** *p* < 0.001.

**Figure 3:**
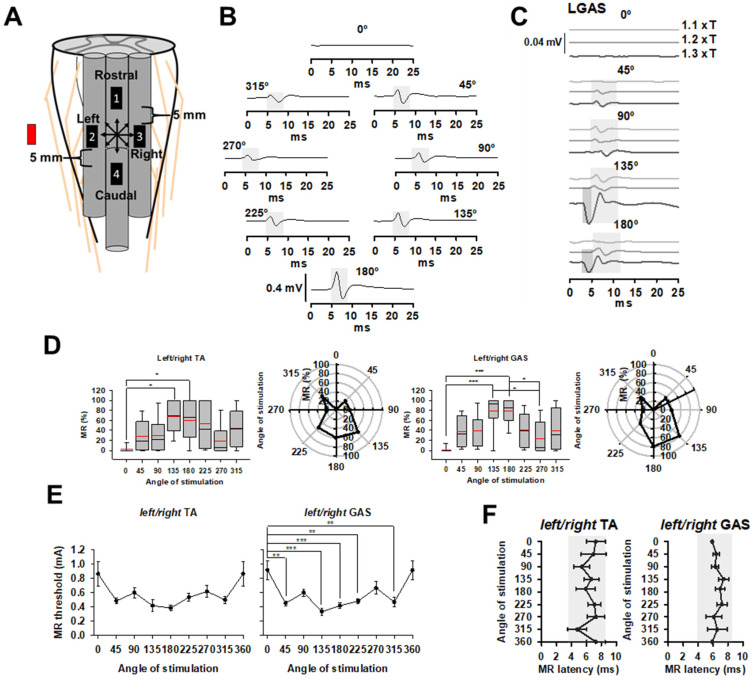
Orientation Selective Epidural Stimulation (OSES). (**A**) A total of 3 contacts of the 4-contact lead were used to deliver OSES every 45° in a clockwise direction. Red rectangle represents the relative location of anode. Arrows indicate the direction of the electric field. (**B**) SEMPs in a representative experiment during OSES in left GAS at 1.2 xT. (c) SEMPs evoked in left GAS at increasing stimulation currents at 1.1 xT, 1.2 xT and 1.3 xT from 0° to 180°. Dark and light gray rectangles highlight ER and MR, respectively. Traces shown in (**B**,**C**) correspond to ten averaged responses. (**D**) Normalized MR amplitudes in bilateral TA (left panel) and GAS (right panel) during OSES delivered at 1.2 xT. Statistical differences were found when comparing 0° vs. 135° and 180° on left/right TA. In left/right GAS, significant differences were found when comparing 0° vs. 135° and 180°, and between 135° and 180° vs. 270°. (**E**) MR thresholds (mean ± SE mA, *n* = 5) during OSES in bilateral TA (left panel) and GAS (right panel), at each angle of stimulation. Statistical differences were found in left/right GAS when comparing 0° vs. 45°, 135°, 180°, 225° and 315°. (**F**) MR latencies across angles of stimulation are shown for bilateral TA (top panel) and GAS (low panel) evoked at 1.2 xT. Values turned out to be in the expected range for MR as depicted by the light gray rectangles. * *p* < 0.05, ** *p* < 0.01, *** *p* < 0.001. (*n* = 5).

Surgery and implantations: Six adult male Sprague Dawley rats (300–350 g body weight) were used in this study. The experimental procedures comply with the guidelines of the National Institute of Health Guide for the Care and Use of Laboratory Animals and are conducted in accordance with protocol approved by the Animal Care Committee at the Mayo Clinic, Rochester, MN. (A00001833-16 Functional Restoration of Movement in a Rodent Model of Paralysis. Approval date: 8 December 2016). The rats were anesthetized by a single urethane dose (1.5 g/kg, IP). A mid-dorsal skin incision was made between T12 and L6 vertebrae and paravertebral muscles were retracted as needed. Partial laminectomies were performed at the T13-L1, L2, and L3-L4 levels. Four Teflon-coated stainless-steel wires (AS632, Cooner Wire, Chatsworth, CA, USA) were tied together and a small notch made in the Teflon-coating (~1.0 mm) on each wire was used as a contact, identified as (1) rostral, (2) left, (3) right and (4) caudal. Distances between contacts in the rostral–caudal direction are depicted in Figure 1A. The whole array was inserted through the T13-L1 window and slid toward the desired location at L2. The bottom wire was routed caudally exiting through the window at L3-L4. A Teflon-coated wire was stripped from the distal part (~1 cm) and was inserted in the paravertebral muscle’s region on the left side and served as a reference in CMES (Figure 1A), as a ground in SSES (Figure 2A), and as reference in OSES (Figure 3A). All exposed tissues were irrigated liberally with warm, sterile saline solution and closed in layers. During testing, the body temperature of the rat was maintained at 36 ± 1 °C by using a water-circulating heating pad and subcutaneous administration of 1.5 mL of warm saline solution (NaCl 0.9%) every two hours. Bipolar needle EMG electrodes (Medtronic, Memphis, TN) were inserted bilaterally into the tibialis anterior (TA) and gastrocnemius (medial or lateral, GAS) muscles and taped on the skin. EMG signals were recorded (4000 Hz), amplified, and filtered (10 to 1000 Hz band-pass) using Lab Chart (AD Instruments).

Stimulation paradigms: 10 consecutive pulses were delivered (pulse width 0.5 ms, 0.5 Hz) in 0.1 mA increments within a range between 0.2 mA and 1.2 mA. Characteristics of EES using the four electrodes array for each protocol are described as follows: (1) CMES was delivered using one contact lead at a time with the reference (Figure 1A), using a single channel stimulator (A-M Systems, Sequim, WA, USA); (2) SSES was performed using the four contact leads of the electrode array in eight combinations as depicted in Figure 2A. SSES was delivered using 8 independent-channels stimulator (STG4008, Multichannel Systems, Reutlingen); (3) OSES was delivered with the same electrode array and stimulator as SSES but by using three contact leads as shown in Figure 3A. OSES was generated with square waveforms as was originally introduced for deep brain stimulation (DBS) in [26] and subsequently utilized in [27]. The relative current amplitudes I1, 2, 3 of the three channels were chosen based on sinusoidal functions with phase offsets of 120°, given by the following:

I1 = I0 sin (Φ), I2 =  I0 sin (Φ  +  120°), I3 =  I0 sin (Φ − 120°).(1)

Here, I0 is the stimulation current amplitude and Φ governs the stimulation angle. The electric field gradient is induced such that the principal direction is defined by the phase step along the sinusoids. The current distribution between the three stimulation electrodes was chosen to generate incremental steps of 45° of reorientation of the electric field gradients on the plane connecting the three electrodes. The angles of stimulation were set such that 0°/180° corresponds to the rostral–caudal direction while −90°/90° corresponds to the left–right direction, respectively. Location of the cathode defines the angle of stimulation. The angle 0° (360°) was defined as the rostral angle of stimulation, parallel to the longitudinal axis of the spinal cord [26]. For each angle of stimulation, ten pulses were delivered as described above. The three EES paradigms: CMES, SSES, and OSES were applied to each animal and are summarized in Table 1. Each experimental session lasted for about 4 h.

Data analysis: For each EES paradigm, comparisons of the SEMPs from each recorded muscle were performed at the different current intensities (Table 1). Early Responses (ER) and Middle Responses (MR) [31,32,33] were manually determined using Clampfit 10.6.22 (Molecular Devices, LLC) as follows: (a) latencies were measured from the beginning of the stimulus artifact considering two window times: 1.5 to 4.5 ms for ER and 4.5 to 10.5 ms for MR; (b) amplitudes of SEMPs were defined as the “peak-to-peak” values. Latencies and amplitudes were averaged (10 responses) for each stimulation current in all recorded muscles. The amplitude of SEMPs was variable among muscles; thus, the averaged amplitudes are reported as the percentage of the maximal response (100%) for MR in a given muscle for each EES paradigm. MR obtained in a range of stimulation between 0.4 mA and 0.8 mA are presented in the results, as MR represents the activation of spinal circuits (mono- or disynaptic reflexes) [31]. Additionally, the range of stimulation (0.4–0.8 mA) was selected due to the fact that at lower stimulation currents MR was absent (i.e., 0.1 mA) and at higher intensities (above 0.8 mA) MR was “fused” with the ER component complicating identification and comparison of MR, and to avoid decrease in MR at higher stimulation currents as previously reported [31,32,33]. Increasing stimulation currents were expressed as x times threshold (xT) for MR.

Statistical analyses: All data are reported as mean ± SE. Normality of data and equal variance were tested with the Shapiro–Wilk and Brown–Forsythe test, respectively. Statistically significant differences were determined using a one-way repeated measures analysis of variance (ANOVA). The Holm–Sidak method was used to perform pairwise multiple comparisons. Data that were not normally distributed were analyzed using the Kruskall–Wallis test and pairwise multiple comparisons were performed using the Tukey test. The criterion level for statistical significance was set at *p* < 0.05 for all analyses. All statistical analysis was performed using SigmaPlot 14.0 (Systat Software, Inc., Chicago, IL, USA). Averaged and normalized amplitudes were determined for right/left TA (RTA, LTA) and GAS (RGAS, LGAS), and then comparisons in these muscles were made according to the stimulation paradigm: for CMES, amplitudes were compared between electrode leads; in SSES paradigm, comparisons between electrode configurations and their reverse polarities were made; finally, amplitudes between angles of stimulation were compared in the OSES paradigm.

## 3. Results

### 3.1. Conventional Monopolar Epidural Stimulation, CMES

First, we evaluated the amplitudes of MR with CMES. Cathodic stimulation was delivered in each of the four contacts at a time: rostral, left, right, and caudal (Figure 1A) at 1.1 xT (0.4 mA), 1.2 xT (0.5 mA) and 1.3 xT (0.6 mA). An anode (red rectangle in Figure 1A) was inserted on the paravertebral muscles on the left. Figure 1B shows an example of averaged SEMPs (10 responses) recorded bilaterally in TA and GAS muscles, evoked at 1.1 xT, 1.2 xT, and 1.3 xT. ER and MR are highlighted in dark and light gray, respectively (Figure 1B). The rostral electrode produced the highest bilateral amplitude responses in both TA and GAS compared to caudal, left and right electrodes. At 1.1 xT no responses were observed when stimulation was applied with the caudal contact in both TA and GAS. Low-amplitude SEMPs at 1.1 xT and 1.2 xT were also observed (Figure 1B). The averaged MR amplitudes for each muscle recorded across contact leads are shown in Figure 1C, when CMES was delivered at 1.1, 1.2 and 1.3 xT (gray, dark gray and black lines, respectively). Because consistent MR was obtained at 1.2 xT, avoiding overlapping with ER, SEMPs amplitudes were used to construct plots as shown in Figure 1D. Higher amplitudes were observed when stimulation was delivered at rostral compared to the caudal, left, and right contacts; however, no significantly differences were found (*p* > 0.05, Figure 1D).

### 3.2. Bipolar Spatial Selective Epidural Stimulation, SSES

Next, we evaluated the effect of SSES. We used the same 4-contact electrode array (Figure 2A) to provide different bipolar electrode configurations and their reversed polarities resulting in 8 configurations (Figure 2B). MR amplitudes across tested muscles were evaluated when SSES was delivered at 0.4 mA (1.1 xT), providing consistent results with MR observed in each combination of the contact leads used for SSES as presented in Figure 2B for a representative experiment (each trace represents 10 averaged responses). Lower stimulation intensities (i.e., 0.2 mA) did not evoke MR in some configurations, while stimulation at 0.6 mA produced overlapped ER and MR responses making it difficult to compare amplitudes. Normalized and averaged MR amplitudes (±SE) evoked during SSES are presented in Figure 2C (*n* = 3). Each bar plot corresponds to a bipolar configuration (black bars, 1, 3, 5, and 7) and their reverse polarity (gray bars 2, 4, 6, and 8). Overall, the maximum amplitudes were observed with configurations 1 and 8, while lower MR amplitudes were found when SSES was delivered with the reversed polarities 2 and 4 for all tested muscles (Figure 2C). All pair-wise comparisons across SSES electrode configurations for each muscle were performed. In LGAS, no statistical differences were found when comparing the electrode configurations and their reversed polarities (*p* > 0.05). Similar results were found for LTA in all configurations (*p* > 0.05) (Figure 2C). For RGAS, a statistical difference was observed when comparing the following configurations: 1 vs. 2 (*p* = 0.002), 1 vs. 3 (*p* = 0.028), 1 vs. 4 (*p* = 0.001), 1 vs. 5 (*p* = 0.002), 1 vs. 6 (*p* = 0.004), and 1 vs. 7 (*p* = 0.018). A similar pattern was found for RTA, and significant differences were found when comparing the following configurations 1 vs. 2, (*p* = 0.002), 1 vs. 4 (*p* = 0.007), 1 vs. 5 (*p* = 0.045), 1 vs. 6 (*p* = 0.047), 2 vs. 3 (*p* = 0.014), 2 vs. 8 (*p* = 0.004), and 4 vs. 8 (*p* = 0.016) (Figure 2C).

A summary of results for SSES, mean (±SE%) and comparisons between configurations 1, 3, 5, and 7 and their reversed polarities (2, 4, 6, and 8) for bilateral GAS and TA are shown in Table 2. Overall, comparison between different bipolar configurations indicates the significant role of orientation of the electrical field on the threshold and amplitude of SEMPs.

### 3.3. Orientation Selective Epidural Stimulation, OSES

Next, we evaluated the effect of OSES generated by the minimal number of electrodes to produce OSES by selecting three out of four available contact leads (see Methods, and [26]). The same 4-contact leads electrode was used in CMES and SSES (Figure 3A). The orientation of the electric field gradient generated with OSES was changed at 45° intervals in a clockwise rotation (Figure 3). Examples of averaged SEMPs (ten averaged responses) in LGAS produced by OSES delivered at 0.4 mA are shown in Figure 3B (MR are highlighted by light gray rectangles). No MR were evoked at 0° (0.00 ± 0.0 mV), while at 180° the highest MR was observed (0.41 ± 0.1 mV). OSES generated at the rest of the angles produced a noticeable MR, measured as peak-to-peak amplitudes: 45° (0.18 ± 0.02 mV), 90° (0.15 ± 0.06 mV); 135° (0.22 ± 0.03 mV), 225° (0.19 ± 0.05 mV), 270° (0.12 ± 0.05 mV) and 315° (0.14 ± 0.06 mV). MR amplitudes were relatively similar during OSES orientation to the right (45°, 90° and 135°) and to the left (225°, 270° and 315°) (Figure 3B). Representative averaged SEMPs in one experiment with OSES delivered at 0°, 45°, 90°, 135° and 180° at three different stimulation intensities (1.1 xT, 1.2 xT, and 1.3 xT) are shown in Figure 3C. Interestingly, no SEMPs were found during OSES delivered at 0°, even at the highest stimulation current tested (1.3 xT). Also, note that OSES at 1.3 xT at 135° and 180° evoked ER superimposed with MR (dark gray and light gray rectangles, respectively). Based on the results above, OSES at 1.2 xT was chosen to analyze MRs. Because no statistical differences were found when comparing left vs. right SEMPs during OSES (TA, *n* = 6, GAS *n* = 5, *p* > 0.05), left/right GAS, and left/right TA MRs were averaged for further analysis. MR normalized amplitudes for left/right TA and GAS (mean ± SE%) evoked at the different angles generated with OSES are shown in Figure 3D. In both TA and GAS, a similar trend was noticeable, with lower amplitudes at 0° (360°) and the highest at 135° and 180° as also shown in polar plots in the same Figure 3D. Only OSES at 135 and 180° was found to be significantly higher compared to 0° (360°) in TA (*p* = 0.016), and in GAS (*p* < 0.001) (Figure 3D). In GAS, OSES at 135° and 180° also exhibited a higher amplitude compared to 270° (*p* = 0.017) (Figure 3D). Averaged MR amplitudes (±SE) across angles of OSES are presented in Table 3. Then, the thresholds of MR depending on the angle of stimulation were expressed as stimulation current (mA) necessary to evoke MR during different angles of OSES and presented in Figure 3E for bilateral TA and GAS. MR thresholds were compared across angles of OSES stimulation for left/right TA (*n* = 6) and GAS (*n* = 5). For left/right TA, no statistical differences were found across angles of stimulation. For left/right GAS, a higher current was delivered to evoke an MR threshold at 0° (360°) compared to 45° (*p* = 0.002); 135° (*p* < 0.001); 180° (*p* < 0.001); 225° (*p* = 0.009) and 315° (*p* < 0.003). Finally, the averaged MR latencies obtained at each angle of stimulation are shown in Figure 3F for left/right TA (*n* = 6) and GAS (*n* = 5), demonstrating the usual range for MR (4–10 ms) highlighted by light gray rectangles. No significant differences in latencies across angles of OSES for TA and GAS were found (*p* > 0.05).

## 4. Discussion

The targeting of specific spinal cord structures with EES is essential for the development of effective clinical protocols for spinal cord neuromodulation. In conventional EES, the electrodes are placed on a dorsal surface of the dura mater that encases the highly conductive cerebral spinal fluid, mediating the electrical field to the main targets, dorsal columns, and dorsal roots [34,35]. In this multilayer system, the selectivity of the target structure activation is limited by the number of electrode contacts, their spatial distribution, and the options to provide flexible adjustment in the orientation of the electrical field. Spatial orientation of the dorsal spinal structures [36], electrical properties of the spinal cord and nerve fibers [37,38], and direction of the electric field across stimulating tissues [35,36] are critical factors determining the effect of EES. Computational modeling of EES suggests that activation of neural structures depends on the orientation of electrical field gradients along the fibers [34], and is directly related to the dorsal root anatomy and the angles between the fibers and the spinal cord axis [35,39,40]. The thick dorsal root fibers are primarily recruited at the lowest EES intensities [41,42], and, particularly, Group Ia/Ib and Group II afferents are the first neural elements depolarized during EES [43,44].

Previously, it was demonstrated that EES applied to the lumbar spinal segments can activate dorsal and ventral roots reflecting the appearance of the motor-evoked responses at different latencies in animals [31,32] and in humans [6,15]. In rats, MR appears in muscles at lower EES current intensities, representing the activation of the low threshold Ia muscle afferents in dorsal roots with latencies between 5 and 10 ms. At higher intensities, EES activates ventral roots, producing a direct (ER) with a shorter latency of about 3–5 ms. The difference in timing of these two responses is primarily due to an intraspinal synaptic delay from dorsal root structures to ventral horn motor neurons [31,32,33]. We further demonstrated that spinal neuroanatomy, and particularly the location and orientation of the dorsal root in relation to the stimulating contacts, is a critical factor in determining the magnitude of motor responses [14] or ECAP [19] as enabled via EES. Based on these findings, we evaluated the segment-specific orientation of the dorsal and ventral roots in humans, proposing a segment-specific stimulation approach [15]. These results emphasize the importance of developing spinal cord spatial-selective protocols.

This study evaluates three different EES modalities by comparison of the amplitudes of the MR. EES modalities were implemented with a four-contact leads electrode array to provide conventional monopolar stimulation CMES, bipolar SSES, and OSES produced by a minimal number of contact leads. With CMES, EES was delivered with one of four monopolar configurations identified as rostral, left, right, and caudal, at a time. MR amplitudes with CMES were related to the position of the stimulating electrode, i.e., EES applied at the rostral contact produced higher MR amplitudes in all muscles compared to the most caudal contact (Figure 1B–D). This further emphasizes the importance of neuroanatomy in terms of the proximity of the electrodes to dorsal roots and segmental location. Bipolar SSES was delivered by combining active contact leads and their reversed polarities (Figure 2A,B). Although MR amplitudes exhibited a similar trend across tested muscles, bipolar configurations 1, and 3 produced higher MR amplitudes compared to their corresponding reverse polarities 2 and 4, respectively. Interestingly, significant differences between electrode configurations were found just in the right GAS and TA, although high variability between polarities was observed (Figure 2C). Variations in MR amplitude are evident with reversed polarities, indicating the importance of the orientation of the electrical field. As in the CMES protocol, higher MR amplitudes were found in SSES when the rostral contact lead was used as a cathode (configuration 1) (Figure 2C).

Differences in MR amplitudes were more evident with OSES, where low MR amplitudes or even absence of MR was observed at 0° (360°), while higher amplitudes were found at 135°, 180° and 225° with a difference up to 60% compared to the rest of the angles of stimulation (Figure 3B–D). With OSES, the distribution of electrical field delivered by three-contact leads vs. common reference electrode (Figure 3) may spread in different directions compared to CMES and SSES. In fact, MR amplitudes during OSES were consistent with the orientation of the dorsal roots, specifically at 135° and 225°, as reported previously for brain stimulation [26]. In this context, OSES represents a new approach to activate specific neural pathways considering the orientation of the selected target fibers.

In general, an efficient targeting of axonal pathways could be accomplished by reorienting the gradients of the electric field using independently controllable channels, an approach that we introduced recently in applications to DBS [26] for efficient target stimulation in different brain areas [45]. With the appropriate independent control of the electrode channels, a two-dimensional pattern of stimulation can be formed, and the electrical fields can be generated to have a directionally dependent intensity, which can be spatially dependent or time dependent. Namely, gradients of the electric fields, dE/dl and dE/dt, could be generated.

Notably, the orientation selective stimulation allows for independent control of the amplitude, frequency, and phase of the current, or voltage, in each channel. Specifically, the amplitude, frequency, or phase in a given channel may be constant or modulated according to a channel-specific function. Using this independent control of the individual electrode channels in the multichannel electrode, based on orientation selectivity, one can generate electromagnetic fields that are capable of stimulating neurons or other cells regardless of their orientation, or can generate spatially selective electromagnetic fields to preferentially stimulate neurons oriented along specific directions. The directionally dependent intensity during OSES can allow for preferential stimulation of neurons oriented along particular directions, such as fiber bundles with anisotropic geometry, or a group of axons oriented predominantly in one direction. This is advantageous to SSES during which the directionality of the propagating electromagnetic wave is strictly governed by the geometry of the contacts having invariant phases. Therefore, OSES provides greater angular selectivity and flexibility for reorienting electric field’s spatial and temporal gradients on a plane or in 3D space. Thus, larger differences in MR amplitudes which were evident with OSES can be attributed to the greater flexibility of this method to generate more efficient stimulation as compared to directional and monopolar stimulations.

To achieve full reorientation flexibility of the electrical field gradients on a plane, a minimum of only 3 independently controllable channels distributed along a triangle is required, while reorientation in space in 3D could be achieved with a minimum of 4-channel electrodes, as we have recently shown for the stimulation of the infralimbic cortex in the rat’s brain [27]. Orientation-selective (OS) DBS was demonstrated to be effective for enhancing activation of specific networks also with multichannel electrodes with independently controlled channels used for human therapy [30]. Specifically, OS activation can be achieved using clinically available DBS leads composed of cylindrical contacts, which enable selectively activating axons parallel and perpendicular to the lead shaft. Notably, independent multiple current sources combined with a segmented lead provide selectivity of stimulation that could be varied within −90° and 90°. This suggests that greater activation of a target oriented perpendicular to a DBS lead could be obtained while avoiding adjacent tracts coursing parallel to the lead shaft, indicating an important path for further developments in pre-clinical and clinical applications. As such, with extensive progress of multielectrode arrays with independently controlled contacts, stimulation in 3D space could be achieved with sufficient flexibility [27].

Similarly, the OS concept could be applied for epidural stimulation of the spinal cord as demonstrated here using rats. The current limitation, however, is that most electrodes used for spinal cord stimulation in clinical practice employ multiple channels that are driven by a single current source, thus limiting the possibility of reorienting the electric field gradient on a plane by changing the current amplitude in each channel separately. Therefore, although allowing steering flexibility (i.e., directing the electrical field in space to the target of interest), the reorientation flexibility of the electrical field spatial gradients is largely limited when using a single current source for all channels. This is also because each channel of the commercially used human leads can be switched only between cathode and anode, and thus the geometrical distribution of the contacts dictates the gradient’s orientation of the electric fields. Because the use of multiple current sources is being increasingly exploited for SCS, the wide availability of such electrodes opens wide opportunities for OSES of the spinal cord for both research purposes and immediate treatment [29].

It should be emphasized that a relatively small group of animals is certainly a limitation of this study. However, considering the total number of responses recorded in four different muscles, we expect that the results are representative, despite the relatively small number of animals used. Additionally, we included animals in which the full protocol was completed with the three stimulation paradigms. In this sense, our results support the key statement of this study and are highly relevant to the field of epidural electrical stimulation. Based on these findings, new stimulation paradigms could be generated to control pain and treat spinal cord injury and other conditions.

In the spinal cord, the specificity of EES is dependent on a multitude of factors such as conductivity of tissues between the electrode and the spinal cord, location and the number of contact leads, and size of the electrode contact surface. A higher density of contact leads and a combination of different spatial modalities might improve specific targeting of the neural elements. For example, cat sacral nerves activity related to lower urinary tract was accomplished with EES electrodes of 16 or 24 contact leads, providing evidence of relatively selective stimulation using one active electrode at a time. “Hot spots” as defined by the authors [46], could be targeted more effectively by spatially selective approaches as the one used in this study. In this study, the small size of the rat’s spinal cord limits the number of contacts that can be positioned over the targeted anatomical region in a practical manner. Future experiments should reveal the feasibility of combining the spatial-orientation modalities in awake animals with SCI to assess the facilitation of motor responses during walking on a treadmill, i.e., improved walking compared to conventional EES.

## 5. Conclusions

The present study, for the first time, compares spatial-selective modalities of EES of the spinal cord. By assessing the variations in SEMPs, we demonstrate that SSES and OSES could provide more selective activation of spinal cord structures depending on chosen configurations or current orientation. The results show that the amplitude of motor potentials in hindlimb muscles depends on the distribution and orientation of the electrical field and varies between applied modalities of stimulation and that spatially selective spinal cord stimulation can tune stimulation with a better motor outcome.

Further advancing this technique could lead to a new level of functional targeting of the spinal cord structures and provide control of chronic pain, restoration of sensorimotor and autonomic functions, and treatment of multiple neurological conditions with spinal cord neuromodulation. Recent advanced stimulators with multiple independently controllable leads and waveforms, e.g., Ilumina3D systems from Boston Scientific are available for EES, thus opening a window of opportunities also for OSES in immediate clinical practice.

## Figures and Tables

**Table 1:** Summary of EES paradigms performed in this study.

EES Paradigm	Characteristics	Pulses
CMES	4 electrodes array, electrical stimulation performed each electrode at a time, 4 configuration, monopolar stimulation.	10 pulses for each stimulation current Range: 0.2–1.2 mA Increments: 0.1 mA
SSES	4 electrodes array, 8 different configurations, bipolar stimulation.	
OSES	Electrical stimulation delivered every 45° in a clockwise rotation using 3 electrodes, 9 different orientations.	

Abbreviations: CMES, Conventional Monopolar Epidural Stimulation; SSES, Spatially Selective Epidural Stimulation; OSES, Oriented-Selective Epidural Stimulation.

**Table 2:** Comparison of MR amplitudes (mean ± SE%, *n* = 3) during SSES in bilateral TA and GAS.

Muscle	Configuration 1	Configuration 2	*p* Value
LGAS	59.09 ± 23.40%	21.83 ± 17.92%	*p* > 0.05
LTA	82.04 ± 13.48%	19.76 ± 19.23%	*p* > 0.05
RGAS	100 ± 2.98%	7.81 ± 2.65%	*p* < 0.001
RTA	100 ± 9.67%	11.36 ± 5.45%	*p* < 0.001
	**Configuration 3**	**Configuration 4**	
LGAS	47.85 ± 20.98%	11.23 ± 3.54%	*p* > 0.05
LTA	69.91 ± 15.78%	20.06 ± 9.94%	*p* > 0.05
RGAS	33.23 ± 17.60%	5.93 ± 3.97%	*p* > 0.05
RTA	82.62 ± 7.85%	22.95 ± 22.40%	*p* > 0.05
	**Configuration 5**	**Configuration 6**	
LGAS	21.81 ± 1.37%	23.28 ± 5.71%	*p* > 0.05
LTA	13.25 ± 5.09%	23.57 ± 10.26%	*p* > 0.05
RGAS	10.75 ± 10.75%	16.51 ± 13.09%	*p* > 0.05
RTA	38.04 ±11.74%	39.21 ± 20.29%	*p* > 0.05
	**Configuration 7**	**Configuration 8**	
LGAS	57.57 ± 23.98%	39.90 ± 30.58%	*p* > 0.05
LTA	52.34 ± 27.37%	88.34 ± 6.12%	*p* > 0.05
RGAS	28.80 ± 16.42%	66.06 ± 21.40%	*p* > 0.05
RTA	54.77 ± 9.23%	93.16 ± 4.48%	*p* < 0.05

**Table 3:** MR amplitudes normalized (%) for TA and GAS during OSES. Values are presented as mean ± SD % (TA *n* = 6; GAS *n* = 5). Comparisons between angles for left/right TA and left/right GAS are represented by symbols.

OSES (Degrees)	MR Amplitude Normalized (Mean ± SD%)	
	Left/Right TA	Left/Right GAS
0 (360)	2.15 ± 5.69 *	1.70 ± 4.82 ^#§^
45	28.33 ± 30.11	37.59 ± 31.47
90	30.32 ± 34.90	39.32 ± 31.05
135	67.18 ± 32.68 *	79.59 ± 24.60 ^#¥^
180	59.90 ± 36.68	78.91 ± 24.60 ^§£^
225	53.97 ± 43.25	40.43 ± 33.50
270	19.24 ± 30.64	23.61 ± 32.64 ^¥£^
315	42.80 ± 36.86	38.69 ± 40.77

* *p* = 0.016, ^#^ *p* < 0.001, ^¥^ *p* = 0.017, ^§^ *p* < 0.001, ^£^ *p* = 0.018.

## Data Availability

The data and codes supporting the findings of this study are available from the authors, upon reasonable request. The data are not publicly available due to requirements of the funding bodies and institutional practices.

## References

[1] Shealy C.N., Mortimer J.T., Reswick J.B. (1967). Electrical Inhibition of Pain by Stimulation of the Dorsal Columns: Preliminary Clinical Report. Anesth. Analg..

[2] Minassian K., Jilge B., Rattay F., Pinter M.M., Binder H., Gerstenbrand F., Dimitrijevic M.R. (2004). Stepping-like Movements in Humans with Complete Spinal Cord Injury Induced by Epidural Stimulation of the Lumbar Cord: Electromyographic Study of Compound Muscle Action Potentials. Spinal Cord.

[3] Harkema S., Gerasimenko Y., Hodes J., Burdick J., Angeli C., Chen Y., Ferreira C., Willhite A., Rejc E., Grossman R.G. (2011). Effect of Epidural Stimulation of the Lumbosacral Spinal Cord on Voluntary Movement, Standing, and Assisted Stepping after Motor Complete Paraplegia: A Case Study. Lancet.

[4] Angeli C.A., Edgerton V.R., Gerasimenko Y.P., Harkema S.J. (2014). Altering Spinal Cord Excitability Enables Voluntary Movements after Chronic Complete Paralysis in Humans. Brain.

[5] Rejc E., Angeli C.A., Atkinson D., Harkema S.J. (2017). Motor Recovery after Activity-Based Training with Spinal Cord Epidural Stimulation in a Chronic Motor Complete Paraplegic. Sci. Rep..

[6] Grahn P.J., Lavrov I.A., Sayenko D.G., Van Straaten M.G., Gill M.L., Strommen J.A., Calvert J.S., Drubach D.I., Beck L.A., Linde M.B. (2017). Enabling Task-Specific Volitional Motor Functions via Spinal Cord Neuromodulation in a Human With Paraplegia. Mayo Clin. Proc..

[7] Chalif J.I., Chavarro V.S., Mensah E., Johnston B., Fields D.P., Chalif E.J., Chiang M., Sutton O., Yong R., Trumbower R. (2024). Epidural Spinal Cord Stimulation for Spinal Cord Injury in Humans: A Systematic Review. J. Clin. Med..

[8] Greiner N., Barra B., Schiavone G., Lorach H., James N., Conti S., Kaeser M., Fallegger F., Borgognon S., Lacour S. (2021). Recruitment of Upper-Limb Motoneurons with Epidural Electrical Stimulation of the Cervical Spinal Cord. Nat. Commun..

[9] Rowald A., Komi S., Demesmaeker R., Baaklini E., Hernandez-Charpak S.D., Paoles E., Montanaro H., Cassara A., Becce F., Lloyd B. (2022). Activity-Dependent Spinal Cord Neuromodulation Rapidly Restores Trunk and Leg Motor Functions after Complete Paralysis. Nat. Med..

[10] Harkema S.J., Wang S., Angeli C.A., Chen Y., Boakye M., Ugiliweneza B., Hirsch G.A. (2018). Normalization of Blood Pressure With Spinal Cord Epidural Stimulation After Severe Spinal Cord Injury. Front. Hum. Neurosci..

[11] Aslan S.C., Legg Ditterline B.E., Park M.C., Angeli C.A., Rejc E., Chen Y., Ovechkin A.V., Krassioukov A., Harkema S.J. (2018). Epidural Spinal Cord Stimulation of Lumbosacral Networks Modulates Arterial Blood Pressure in Individuals With Spinal Cord Injury-Induced Cardiovascular Deficits. Front. Physiol..

[12] Herrity A.N., Williams C.S., Angeli C.A., Harkema S.J., Hubscher C.H. (2018). Lumbosacral Spinal Cord Epidural Stimulation Improves Voiding Function after Human Spinal Cord Injury. Sci. Rep..

[13] Shah P.K., Lavrov I. (2017). Spinal Epidural Stimulation Strategies: Clinical Implications of Locomotor Studies in Spinal Rats. Neuroscientist.

[14] Cuellar C.A., Mendez A.A., Islam R., Calvert J.S., Grahn P.J., Knudsen B., Pham T., Lee K.H., Lavrov I.A. (2017). The Role of Functional Neuroanatomy of the Lumbar Spinal Cord in Effect of Epidural Stimulation. Front. Neuroanat..

[15] Mendez A., Islam R., Latypov T., Basa P., Joseph O.J., Knudsen B., Siddiqui A.M., Summer P., Staehnke L.J., Grahn P.J. (2021). Segment-Specific Orientation of the Dorsal and Ventral Roots for Precise Therapeutic Targeting of Human Spinal Cord. Mayo Clin. Proc..

[16] Krupa P., Siddiqui A.M., Grahn P.J., Islam R., Chen B.K., Madigan N.N., Windebank A.J., Lavrov I.A. (2022). The Translesional Spinal Network and Its Reorganization after Spinal Cord Injury. Neuroscientist.

[17] Malone I.G., Nosacka R.L., Nash M.A., Otto K.J., Dale E.A. (2021). Electrical Epidural Stimulation of the Cervical Spinal Cord: Implications for Spinal Respiratory Neuroplasticity after Spinal Cord Injury. J. Neurophysiol..

[18] Minassian K., Persy I., Rattay F., Dimitrijevic M.R., Hofer C., Kern H. (2007). Posterior Root–Muscle Reflexes Elicited by Transcutaneous Stimulation of the Human Lumbosacral Cord. Muscle Nerve.

[19] Verma N., Romanauski B., Lam D., Lujan L., Blanz S., Ludwig K., Lempka S., Shoffstall A., Knudson B., Nishiyama Y. (2023). Characterization and Applications of Evoked Responses during Epidural Electrical Stimulation. Bioelectron. Med..

[20] Shah P.K., Sureddi S., Alam M., Zhong H., Roy R.R., Edgerton V.R., Gerasimenko Y. (2016). Unique Spatiotemporal Neuromodulation of the Lumbosacral Circuitry Shapes Locomotor Success after Spinal Cord Injury. J. Neurotrauma.

[21] Nandra M.S., Lavrov I.A., Edgerton V.R., Tai Y.-C. A Parylene-Based Microelectrode Array Implant for Spinal Cord Stimulation in Rats. Proceedings of the 2011 IEEE 24th International Conference on Micro Electro Mechanical Systems.

[22] Gad P., Choe J., Nandra M.S., Zhong H., Roy R.R., Tai Y.-C., Edgerton V.R. (2013). Development of a Multi-Electrode Array for Spinal Cord Epidural Stimulation to Facilitate Stepping and Standing after a Complete Spinal Cord Injury in Adult Rats. J. Neuroeng. Rehabil..

[23] Rodger D., Fong A., Li W., Ameri H., Ahuja A., Gutierrez C., Lavrov I., Zhong H., Menon P., Meng E. (2008). Flexible Parylene-Based Multielectrode Array Technology for High-Density Neural Stimulation and Recording. Sens. Actuators B Chem..

[24] Dombovy-Johnson M.L., D’Souza R.S., Ha C.T., Hagedorn J.M. (2022). Incidence and Risk Factors for Spinal Cord Stimulator Lead Migration With or Without Loss of Efficacy: A Retrospective Review of 91 Consecutive Thoracic Lead Implants. Neuromodul. Technol. Neural Interface.

[25] Minassian K., Hofstoetter U., Tansey K., Mayr W. (2012). Neuromodulation of Lower Limb Motor Control in Restorative Neurology. Clin. Neurol. Neurosurg..

[26] Lehto L.J., Slopsema J.P., Johnson M.D., Shatillo A., Teplitzky B.A., Utecht L., Adriany G., Mangia S., Sierra A., Low W.C. (2017). Orientation Selective Deep Brain Stimulation. J. Neural Eng..

[27] Gureviciene I., Laakso H., Narvaez O., Paasonen E., Lehto L., Gurevicius K., Mangia S., Michaeli S., Gröhn O., Sierra A. (2023). Orientation Selective Stimulation with Tetrahedral Electrodes of the Rat Infralimbic Cortex to Indirectly Target the Amygdala. Front. Neurosci..

[28] Wu L., Canna A., Narvaez O., Ma J., Sang S., Lehto L.J., Sierra A., Tanila H., Zhang Y., Gröhn O. (2022). Orientation Selective DBS of Entorhinal Cortex and Medial Septal Nucleus Modulates Activity of Rat Brain Areas Involved in Memory and Cognition. Sci. Rep..

[29] Canna A., Lehto L.J., Wu L., Sang S., Laakso H., Ma J., Filip P., Zhang Y., Gröhn O., Esposito F. (2021). Brain fMRI during Orientation Selective Epidural Spinal Cord Stimulation. Sci. Rep..

[30] Slopsema J.P., Peña E., Patriat R., Lehto L.J., Gröhn O., Mangia S., Harel N., Michaeli S., Johnson M.D. (2018). Clinical Deep Brain Stimulation Strategies for Orientation-Selective Pathway Activation. J. Neural Eng..

[31] Gerasimenko Y.P., Lavrov I.A., Courtine G., Ichiyama R.M., Dy C.J., Zhong H., Roy R.R., Edgerton V.R. (2006). Spinal Cord Reflexes Induced by Epidural Spinal Cord Stimulation in Normal Awake Rats. J. Neurosci. Methods.

[32] Lavrov I., Gerasimenko Y.P., Ichiyama R.M., Courtine G., Zhong H., Roy R.R., Edgerton V.R. (2006). Plasticity of Spinal Cord Reflexes After a Complete Transection in Adult Rats: Relationship to Stepping Ability. J. Neurophysiol..

[33] Lavrov I., Courtine G., Dy C.J., Van Den Brand R., Fong A.J., Gerasimenko Y., Zhong H., Roy R.R., Edgerton V.R. (2008). Facilitation of Stepping with Epidural Stimulation in Spinal Rats: Role of Sensory Input. J. Neurosci..

[34] Coburn B., Sin W.K. (1985). A Theoretical Study of Epidural Electrical Stimulation of the Spinal Cord Part I: Finite Element Analysis of Stimulus Fields. IEEE Trans. Biomed. Eng..

[35] Struijk J.J., Holsheimer J., Boom H.B.K. (1993). Excitation of Dorsal Root Fibers in Spinal Cord Stimulation: A Theoretical Study. IEEE Trans. Biomed. Eng..

[36] Holsheimer J., Struijk J.J. (1991). How Do Geometrie Factors Influence Epidural Spinal Cord Stimulation?. Stereotact. Funct. Neurosurg..

[37] Barolat G. (1998). Epidural Spinal Cord Stimulation: Anatomical and Electrical Properties of the Intraspinal Structures Relevant to Spinal Cord Stimulation and Clinical Correlations. Neuromodul. Technol. Neural Interface.

[38] Holsheimer J. (2002). Which Neuronal Elements Are Activated Directly by Spinal Cord Stimulation. Neuromodul. Technol. Neural Interface.

[39] Ladenbauer J., Minassian K., Hofstoetter U.S., Dimitrijevic M.R., Rattay F. (2010). Stimulation of the Human Lumbar Spinal Cord With Implanted and Surface Electrodes: A Computer Simulation Study. IEEE Trans. Neural Syst. Rehabil. Eng..

[40] Holsheimer J. (1998). Concepts and Methods in Neuromodulation and Functional Electrical Stimulation: An Introduction. Neuromodul. Technol. Neural Interface.

[41] Rattay F. (1999). The Basic Mechanism for the Electrical Stimulation of the Nervous System. Neuroscience.

[42] Rattay F., Minassian K., Dimitrijevic M. (2000). Epidural Electrical Stimulation of Posterior Structures of the Human Lumbosacral Cord: 2. Quantitative Analysis by Computer Modeling. Spinal Cord.

[43] Capogrosso M., Wenger N., Raspopovic S., Musienko P., Beauparlant J., Bassi Luciani L., Courtine G., Micera S. (2013). A Computational Model for Epidural Electrical Stimulation of Spinal Sensorimotor Circuits. J. Neurosci..

[44] Moraud E.M., Capogrosso M., Formento E., Wenger N., DiGiovanna J., Courtine G., Micera S. (2016). Mechanisms Underlying the Neuromodulation of Spinal Circuits for Correcting Gait and Balance Deficits after Spinal Cord Injury. Neuron.

[45] Lehto L.J., Filip P., Laakso H., Sierra A., Slopsema J.P., Johnson M.D., Eberly L.E., Low W.C., Gröhn O., Tanila H. (2018). Tuning Neuromodulation Effects by Orientation Selective Deep Brain Stimulation in the Rat Medial Frontal Cortex. Front. Neurosci..

[46] Jantz M.K., Gopinath C., Kumar R., Chin C., Wong L., Ogren J.I., Fisher L.E., McLaughlin B.L., Gaunt R.A. (2022). High-Density Spinal Cord Stimulation Selectively Activates Lower Urinary Tract Nerves. J. Neural Eng..

